# 2-Aminoethoxydiphenyl borate activates the mechanically gated human KCNK channels KCNK 2 (TREK-1), KCNK 4 (TRAAK), and KCNK 10 (TREK-2)

**DOI:** 10.3389/fphar.2013.00063

**Published:** 2013-05-15

**Authors:** Leopoldo Beltrán, Madeline Beltrán, Ainhara Aguado, Günter Gisselmann, Hanns Hatt

**Affiliations:** Department of Cell Physiology, Ruhr-University-BochumBochum, Germany

**Keywords:** 2-APB, KCNK channels, TREK, *Xenopus oocytes*, two-electrode voltage clamp

## Abstract

Two-pore domain K^+^ (KCNK, K_2_P) channels underlie the “leak” (background) potassium conductance in many types of excitable cells. They oppose membrane depolarization and cell excitability. These channels have been reported to be modulated by several physical and chemical stimuli. The compound 2-aminoethoxydiphenyl borate (2-APB) was originally described as an inhibitor of IP_3_-induced Ca^2+^ release but has been shown to act as either a blocker or an activator for several ion channels. Here, we report the effects of this compound on members of the TREK (TWIK related K^+^ channels) subfamily of human KCNK channels. We injected *Xenopus laevis* oocytes with cRNAs (complementary RNAs) encoding several KCNK channels and measured their response using the two-electrode voltage clamp technique. 2-APB was found to be an effective activator for all members of the TREK subfamily (hKCNK2, hKCNK4, and hKCNK10), with the highest efficacy in hKCNK10. We also found that 2-APB was able to activate these channels in cell-excised patches of HEK293 (human embryonic kidney 293) cell transfected with hKCNK4 or hKCNK10, demonstrating direct activation. TREK channels are widely expressed in the central nervous system and peripheral tissues, where they play roles in several key processes. However, little is known regarding their pharmacology; therefore, the identification of a common, stable and inexpensive agonist should aid further investigations of these channels. Additionally, 2-APB has been used to study native receptors in cell systems that endogenously express members of the TREK subfamily (e.g., rat dorsal root ganglia); our results thus warn against the use of 2-APB at high concentrations in these systems.

## INTRODUCTION

KCNK channels (also known as K_2P_, for two-pore domain potassium channels) are potassium selective channels that tend to be constitutively open. Most of them behave as outward rectifiers under physiological K^+^ concentrations. Additionally, they also behave in an almost voltage-independent manner (Enyedi and Czirják, 2010). Because of these characteristics, they are considered the main channels responsible for the leak potassium current that helps maintain the resting membrane potential. This leak current exerts control over neuronal excitability by shaping the duration, frequency, and amplitude of action potentials (Goldstein et al., 2001). An increased K^+^ leak current stabilizes the cell at hyperpolarized voltages below the firing threshold, whereas leak suppression permits or facilitates depolarization. In this manner, KCNK channels play a role in such diverse processes as metabolic regulation, apoptosis, thermoperception, and chemoperception (Lotshaw, 2007).

The mechanically gated TREK (for TWIK related K^+^ channels) subfamily of KCNK channels is composed of three members: TREK-1 (KCNK2), TRAAK (KCNK4), and TREK-2 (KCNK10) with several functional splice variants reported for hTREK-1 and hTREK-2 (Gu et al., 2002; Xian Tao et al., 2006). Although members of the TREK subfamily differ slightly in their electrophysiological properties, they show similar characteristics, such as mechano- and thermosensitivity. They also present overlapping pharmacological patterns, characterized by a mild sensitivity to standard potassium channel blockers such as quinidine and Ba^2+^ and sensitivity to volatile anesthetics like halothane (Lotshaw, 2007). TREK channels are widely expressed in both the peripheral and the central nervous system (CNS; Medhurst et al., 2001). Recent studies with TREK^-/-^ mice have demonstrated that TREK channels are necessary for appropriate mechano- and thermoperception (Alloui et al., 2006; Noël et al., 2009).

2-Aminoethoxydiphenyl borate (2-APB) was originally described as a membrane-permeable modulator of Ins(1,4,5)P_3_-induced Ca^2+^ release (Maruyama et al., 1997). Although this function has been questioned, 2-APB is agreed to inhibit the store-operated current observed after depletion of ER Ca^2+^ stores (Dobrydneva and Blackmore, 2001); it also blocks certain transient receptor potential (TRP) channels belonging to the canonical (TRPC), and melastatin (TRPM) subfamilies (Hu et al., 2004) and certain voltage-gated potassium channels present in *Limulus* ventral photoreceptors (Wang et al., 2002). It is also a common activator for the TRPV1 (transient receptor potential vanilloid 1), TRPV2, and TRPV3 channels (Hu et al., 2004). Interestingly, the perception of noxious heat, in which TRPV1 and TRPV2 play a key role, has been shown to be modulated or fine-tuned by KCNK2 and KCNK4 (Noël et al., 2009). Both the TREK and TRPVs subfamilies have been shown to be coexpressed at the neuronal level in the rat trigeminal ganglia (Yamamoto et al., 2009). With this background in mind, we addressed the question of whether 2-APB has an effect on human KCNK channels of the TREK subfamily. We found that 2-APB activates all members of this subfamily in a dose-dependent manner and has the greatest effect on hKCNK10.

## EXPERIMENTAL PROCEDURES

### MOLECULAR BIOLOGY

hKCNK4 cloned into pBluescript SK+, hCKNK10 cloned into pCR4-TOPO and hKCNK2 cloned into pDNR-dual were purchased from Imagenes (Berlin, Germany). hKCNK2 and hKCNK10 cDNA inserts were afterward subcloned into pSGEM for electrophysiological recordings in *Xenopus* oocytes. The plasmids were linearized with *Not*I** for pBluescript SK+ and* Pac*I for pSGEM, providing linear cDNA templates for *in vitro* transcription. All three hKCNK inserts were subsequently subcloned into pCDNA3 for electrophysiological recordings in human embryonic kidney 293 (HEK293) cells.

hKCNK3 cloned into the expression vector pIRES-CD8 was a gift from Dr. Fabrice Duprat (Institut National de la Santé et de la Recherche Médicale, Antipolis, France). hKCNK18 cloned into the plasmid pEXO was a gift from Professor Dr. P. Enyedi (Semmelweis University, Budapest, Hungary). hKNCK9 cloned into the expression vector pCR4-TOP, was purchased from Imagenes (Berlin, Germany). The plasmids were linearized with the restriction enzymes *BamH*I,* Xho*I, and *Ssp*I, respectively, and then used as templates for *in vitro* transcription.

Capped RNAs were synthesized in the presence of capping analog m7G(5^′^)ppp(5^′^)G using the AmpliCap-T7 Message Maker kit (Epicentre, Madison, WI, USA). Complementary RNA (cRNA) was dissolved in nuclease-free water to give a final concentration of 500 ng/μL.

### *Xenopus laevis* OOCYTE PREPARATION

Complementary RNAs were expressed in *X. laevis* oocytes essentially as described by Vogt-Eisele et al. (2007). Briefly, mature female *X. laevis* frogs were anesthetized with 0.15% MS-222, surgery was performed according to standard methods, and the extracted ovarian lobes were placed into Ca^2+^-free Barth’s solution containing collagenase type II (Worthington Biochemical Corporation) and incubated for 90 min on a shaker at 40 rpm (room temperature). After that time, healthy stage V and VI oocytes were selected for injection with cRNA coding for the proteins of interest; 30 nL of cRNA (100 ng/μL) for each channel was injected using a Microinjector Micro 4 TM (World Precision Instruments). After injection, the oocytes were incubated in ND96 (96 mM NaCl; 2 mM KCl; 1.8 mM CaCl_2_; 1 mM MgCl_2_; 2.5 mM sodium pyruvate 3 mM, 4-(2-hydroxyethyl)-1-piperazineethanesulfonic acid (HEPES) 10 mM, pH 7.2), and measurements were performed 24–72 h after injection.

The oocytes were placed in a 100 μL chamber perfused with frog Ringer’s solution (115 mM NaCl; 2.5 mM KCl; 1.8 mM CaCl_2_, and 10 mM HEPES pH 7.2); this solution was also used to dissolve the applied chemicals. For recording, a Turbo Tec-03x (npi electronic GmbH) amplifier was used. Borosilicate glass capillaries were pulled with a Kopf vertical pipette puller. For the voltage clamp, the pulled glass capillaries were filled with 3 M KCl.

### TWO-ELECTRODE VOLTAGE CLAMP MEASUREMENTS

A ramp series protocol was used to evaluate the activity of the channels. The voltage ramps consisted of a starting constant voltage of -100 mV for 300 ms, followed by a ramp to +50 mV in 700 ms, then a constant voltage at +50 mV for 300 ms, and a final constant voltage at -40 mV for 300 ms. The time interval between the ramps was of 2 s. In order to evaluate the effect of a substance on the basal current, we took the average of the currents registered during the last 30 ms of the +50 mV constant from the three ramps that showed the maximal response to the applied substance and divided it by the average obtained from three ramps prior to the application.

The data were collected using the *Cellworks Reader 3.7* software (npi instruments, Germany) and analyzed with *Clampfit v10.2.0.14* (MDS Analytical Technologies). Curve fitting was performed using the Hill equation (Sigmaplot 8, Systat software inc.). Data are expressed as means ±SEM (standard error of the mean). A paired *t*-test was used to evaluate the statistical significance of the results, and *P* < 0.05 was considered statistically significant and is marked with one star, *P* < 0.01 is marked with two stars, and *P* < 0.001 is marked with three stars.

### CELL CULTURE

Human embryonic kidney 293 cells were grown in Dulbecco’s modified Eagle’s medium containing 10% fetal bovine serum, 2 mM L-glutamine, and 100 μg/mL penicillin/streptomycin (Invitrogen, Karlsruhe, Germany) at 37°C in a humidity-controlled incubator with 5% CO_2_.

### TRANSIENT EXPRESSION OF HUMAN KCNK2, KCNK4, AND KCNK10 IN HEK293 CELLS

For transient expression of human KCNK2, KCNK4, and KCNK10 in HEK293 cells, we used the recombinant expression plasmid pCDNA3. HEK293 cells were transiently transfected (8 μg of hKCNK4 cDNA in pCDNA3 per dish) in 35-mm dishes (Falcon, BD Bioscience, Heidelberg, Germany) using the CaP-precipitation method described previously by Dörner et al. (2007). These cells were cotransfected with pIRES-EGFP (1 μg per dish), which served as a transfection marker. All recordings were performed 18–24 h after transfection.

### ELECTROPHYSIOLOGY IN HEK293 CELLS

Recordings were performed using the whole-cell and inside-out modes of the patch-clamp technique. Cells were maintained in an extracellular recording solution containing (in mM) 140 NaCl, 5 KCl, 2 MgCl_2_, 2 CaCl_2_, 10 HEPES, and 10 glucose, pH 7.4. Patch electrodes were pulled from borosilicate glass (1.2 mm OD × 1.17 mm ID; Harvard apparatus, Edenbridge, Kent, UK) and fire polished to 4–6 MΩ tip resistance using a horizontal pipette puller (Zeitz Instruments, Munich, Germany) in order to obtain a patch of approximately 1–2 μm diameter.

For recordings, the pipette solution contained (in mM) 140 KCl, 1 MgCl_2_, and 10 HEPES, pH 7.4. All recordings were performed at room temperature with an EPC7 amplifier (List-Medical Electronic, Darmstadt, Germany). For whole-cell patch-clamp recordings, a step protocol consisting of a holding potential of -60 mV, followed by 20 mV steps of 400 ms duration from -110 mV to +50 mV was used. Because the current registered at the last two steps continued to increase and never presented a stable baseline, the last 50 ms of the +10 mV step were selected in order to build a dose–response curve. Cell-excised patches were evaluated in the inside-out configuration, at a holding potential of 0 mV. Data were acquired using Pulse software (HEKA, Lambrecht, Germany). All data are expressed as means ±SEM.

### CHEMICALS

2-Aminoethoxydiphenyl borate and dimethyl sulfoxide (DMSO) were purchased from Sigma-Aldrich (Munich, Germany). Stock solutions of 100 mM and 300 mM 2-APB diluted in DMSO were made and stored at -20°C; the latter was used for preparing solutions containing 3 and 10 mM 2-APB.

## RESULTS

### 2-APB ACTIVATES ALL MEMBERS OF THE TREK SUBFAMILY

**FIGURE 1:**
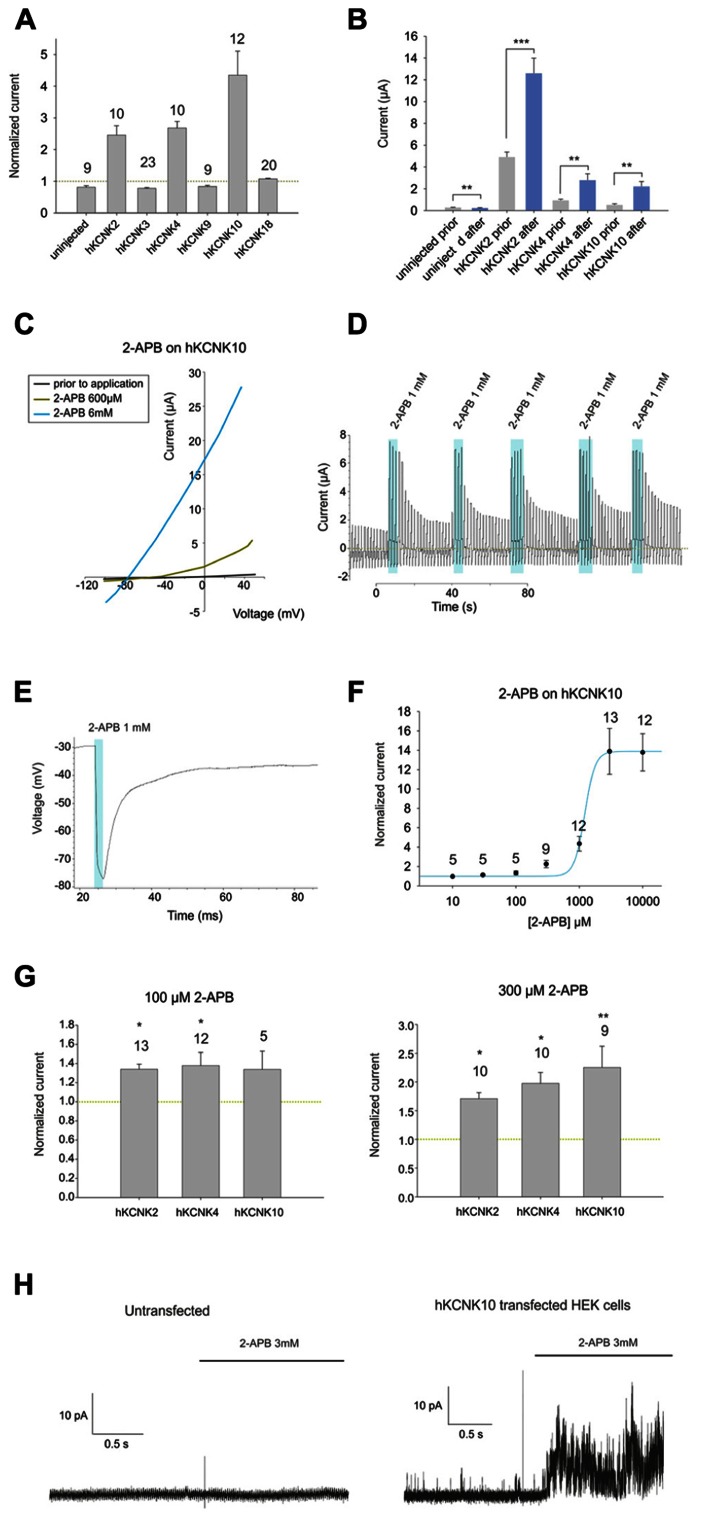
**(A)** Effect of 1 mM 2-APB on (from left to right) uninjected Xenopus oocytes and oocytes injected with cRNA coding for hKCNK2, hKCNK3, hKNCK4, hKCNK9, hKCNK10, and hKCNK18. For each group, the current registered at the final 50 ms of the +50 mV constant (see experimental procedures for a description of the ramp protocol used) was normalized to the current registered prior to the application of 2-APB (dotted line). All data are expressed as means ±SEM. Number of cells in each experiment is indicated above the bars. **(B)** Absolute currents registered in uninjected *Xenopus* oocytes and oocytes injected with cRNA coding for hKCNK2, hKCNK4, and hKNCK10, before and after the application of 1 mM 2-APB. **(C)** Representative I-V trace of a *Xenopus* oocyte expressing hKCNK10 before (black line), and after the application of 2-APB at 600 μM (green line), and 6 mM (blue line). **(D)** Ramp series measurement of a *Xenopus* oocyte expressing hKCNK10 that was repeatedly exposed to 1 mM 2-APB. The dotted green line represents zero current. **(E)** 1 mM 2-APB reversibly hyperpolarizes a *Xenopus* oocyte expressing hKCNK10. **(F) **Dose–response curve fitted to the 4-parameter Hill equation (*n* indicated above the points). **(G)** Effect of 2-APB at 100 μM (left) and 300 μM (right) on *Xenopus* oocytes** injected with cRNA coding for hKCNK2, hKNCK4, and hKCNK10. Data were normalized to the current registered prior to the application of 2-APB (dotted line). All data are expressed as means ±SEM. Number of cells in each experiment is indicated above the bars. **(H)** Inside-out patch-clamp recordings of cell-excised patches from untransfected HEK293 cells (left) and HEK293 cells transfected with hKCNK10 (right) before and after exposure to 3 mM 2-APB.

First, we studied the effect of 1 mM 2-APB on *X. laevis* oocytes injected with cRNAs coding for each member of the TREK subfamily (KCNK2, KCNK4, and KCNK10), two members of the TASK subfamily (KCNK3 and KCNK9), the only member of the TRESK subfamily (KCNK18) and a control group of non-injected oocytes (see **Figure 1A**). Oocytes injected with KCNK18 were not affected by 2-APB, whereas those injected with either member of the TASK subfamily were mildly inhibited by 2-APB. The currents presented by uninjected oocytes were negligible in comparison with the currents of oocytes injected with any of the aforementioned hKCNK channels; however, these currents were mildly but significantly inhibited by 1 mM 2-APB, reducing their basal activity to 70.3% (**Figure 1B**). 2-APB, however, increased the basal activity of hKCNK2, hKCNK4, and hKCNK10 by factors of 2.22 ± 0.2, 2.68 ± 0.2, and 4.35 ± 0.8, respectively.

### 2-APB ACTIVATES hKCNK10 CHANNELS IN A DOSE-DEPENDENT MANNER

We then proceeded to study in detail the effect of 2-APB on hKCNK10. hKCNK10, also known as TREK-2, is expressed throughout the human CNS. It is one of the major background K^+^ channels in dorsal root ganglion neurons (Kang and Kim, 2006), and it is also expressed in the kidneys, spleen, and small intestine (Medhurst et al., 2001). 2-APB produced a dose-dependent (**Figure 1C**) and reversible activation of hKCNK10 that was unaltered after repeated applications (**Figure 1D**). Consequently with this, when we recorded the membrane potential of oocytes expressing hKCNK10, we observed that 1 mM 2-APB produced a strong and quick hyperpolarizing effect that reversed almost completely after washout (**Figure 1E**). The dose–response curve gave an EC_50_ of 1.22 ± 0.39 mM; the maximal effect (seen at around 3 mM) was a 14-fold increase in the basal current (**Figure 1F**). Lower concentrations, however, also led to significant changes in the basal activity of hKCNK10, as this was increased by factors of 1.33 ± 0.2 and 2.25 ± 0.4 by the application of 100 μM and 300 μM, respectively (**Figure 1G**). Because the highest concentration of 2-APB that was evaluated required a concentration of the vehicle compound DMSO higher than 1% (3.3% DMSO for 10 mM 2-APB), we evaluated the effect of this vehicle at its highest concentration used and compared this effect with both the basal current prior to application and basal current after the application of the same solution containing 2-APB (**Figure 3B**). We found no significant difference in the basal current prior and after the application of 3.3% DMSO for 10 s; this was true for both injected and uninjected oocytes (**Figure 3A**).

To determine whether this effect depended on the presence of any cytosolic factor, we proceeded to transfect HEK293 cells with hKCNK10 and measured cell-excised patches in the inside-out configuration, exposing them to 3 mM 2-APB (1% DMSO). Untransfected HEK293 showed no significant response to 3 mM 2-APB (**Figure 1H**, left). Transfected HEK293 cells showed open channel noise, as previously described (Bang et al., 2000), which was markedly increased after the application of 3 mM 2-APB (**Figure 1H**, right), demonstrating the direct nature of the interaction.

### 2-APB ACTIVATES hKCNK2 AND hKCNK4 CHANNELS IN A DOSE-DEPENDENT MANNER

We also investigated the effect of 2-APB on the other two members of the TREK subfamily. These are hKCNK2 (hTREK-1), which is the most thoroughly studied KCNK channel, and hKCNK4 (hTRAAK), which received its name because of its sensitivity to arachidonic acid, though it is also sensitive to other polyunsaturated fatty acids. Both channels are active at physiological body temperatures and have been shown to be necessary for appropriate thermoperception (Noël et al., 2009). Like KCNK10, they are expressed in both the central and peripheral human nervous system (Medhurst et al., 2001).

**FIGURE 2:**
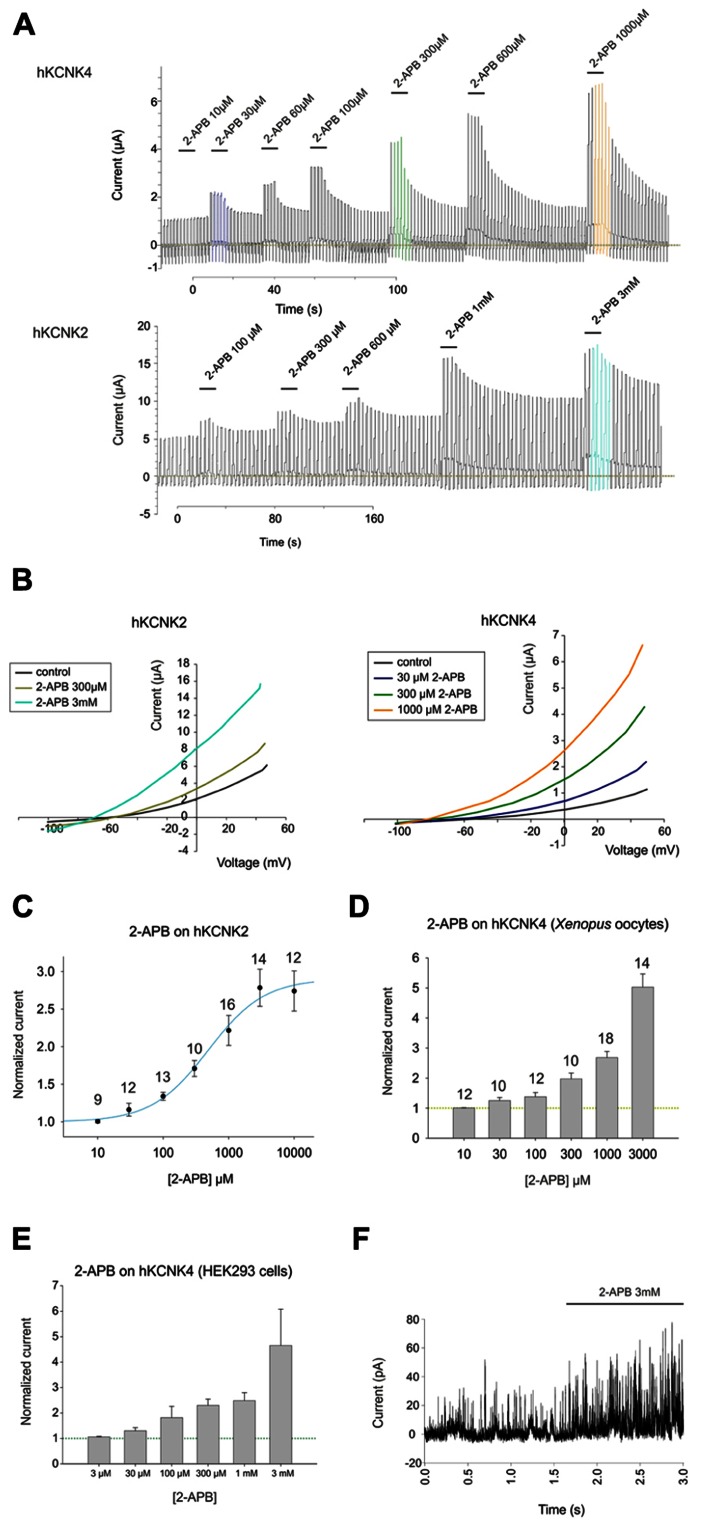
**(A)** Representative traces showing the effect of increasing concentrations of 2-APB on hKCNK4-expressing (top) and hKCNK2-expressing (bottom) *Xenopus* oocytes subjected to the ramp series protocol described in the experimental procedures. **(B)** Representative I–V traces for the excitatory effect of increasing concentrations of 2-APB on oocytes expressing hKCNK2 (left) and hKCNK4 (right). **(C)** Dose–response curve showing the effects of 2-APB on hKCNK2 and fitted to the 4-parameter Hill equation (*n* indicated above the points). **(D)** Dose–response relationship showing the effect of 2-APB on hKCNK4 (*n* indicated above the points). **(E)** Dose–response relationship showing the effect of 2-APB on hKCNK4 (*n* = 3–5 HEK293 cells). **(F)** Inside-out patch-clamp recordings of cell-excised patches from hKCNK4-expressing HEK293 cells before and after exposure to 3 mM 2-APB.

**FIGURE 3:**
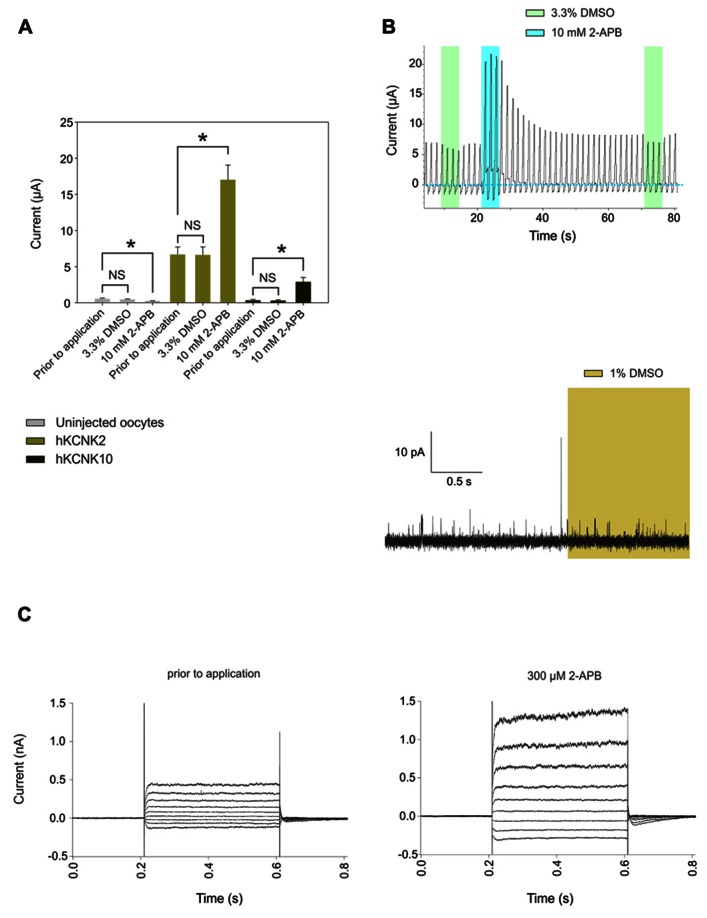
**(A)** Comparison of the effects of applying 100 μL frog Ringer’s solution containing 3. 3% DMSO vs. applying the same solution plus 10 mM 2-APB to uninjected oocytes (gray bars) or oocytes injected with cRNA coding for hKCNK2 (light green bars) and hKCNK10 (dark green bars) (*n* = 3–5 oocytes/group). The time of exposure was approximately 10 s. **(B)** Top: Representative voltage clamp recording from an oocyte expressing hKCNK2 exposed to vehicle solution (green rectangles) or the same solution plus 10 mM DMSO (blue rectangles). Bottom: Representative voltage clamp recording from an inside-out patch-clamp of a HEK293 cell expressing hKCNK10, before and after exposure to 1% DMSO (light green rectangle). **(C)** hKCNK4 currents recorded from a transfected HEK293 cell before (right) and after (left) the application of 300 μM 2-APB. Currents were elicited by voltage pulses from -110 to +50 mV in 20 mV steps, 400 ms in duration, from a holding potential of -60 mV.

2-Aminoethoxydiphenyl borate induced dose-dependent and partially reversible activation of hKCNK2 and hKCNK4 in *X. laevis* oocytes expressing these channels (see **Figure 2A**). As for hKCNK10, 2-APB elicited more pronounced activation of these two channels at potentials above the K_rev_ (**Figure 2B**). 2-APB had an EC_50_ of 486 ± 135 μM for hKCNK2 (**Figure 2C**); however, an EC_50_ value for hKCNK4 could not be determined, although this channel was clearly activated in a dose-dependent manner by 2-APB in both *X. laevis* oocytes (**Figure 2D**) and transfected HEK293 cells (**Figures 2E** and **3C** for original trace). Like hKCNK10, hKCNK4 also showed a marked increase in basal activity when treated with 2-APB in inside-out excised patches from transfected HEK293 cells, suggesting that this effect is independent of cytosolic factors (**Figure 2F**).

## DISCUSSION

Members of the TREK subfamily of KCNK channels are widely expressed in the CNS (Medhurst et al., 2001), where they have been shown to play a key role in several processes ranging from long-term depression to neuroprotection (Heurteaux et al., 2006; Kim et al., 2011). This makes them valuable targets for pharmacological research; however, little is known regarding their pharmacology, and common and stable activators are lacking. Here we have showed that the membrane-permeable substance 2-APB behaves as a highly effective activator for all the members of the mechano-gated TREK subfamily of human KCNK channels. 2-APB must now be regarded as a pharmacological tool for the study of members of the TREK subfamily. Also, its pharmacological characteristics might be useful for drug design. Interestingly, we also found that 2-APB can block certain unidentified endogenous channel(s) in *X. laevis* oocytes. This effect should be taken into consideration when using this system to evaluate 2-APB at high concentrations, especially when characterizing the effects of 2-APB on heterologously expressed channels (e.g., TRPC and TRPM channels).

The basal activity of hKCNK10 was increased up to 2.3- and 14-fold when exposed to 300 μM and 3 mM 2-APB, respectively. This is expected to significantly inhibit cell excitability, as observed in the *Xenopus* heterologous system, in which 1 mM 2-APB induced a hyperpolarizing change in the membrane potential of around -50 mV. It would be interesting to study the effect of this substance on native systems, e.g., trigeminal neurons. 2-APB also had considerable effects on the other members of the TREK subfamily, leading to a fourfold increase in their basal activities. However, as stated, 2-APB acts upon a plethora of other proteins. For example, it activates members of the TRPV subfamily, inhibits members of the TRPC subfamily, and blocks sarco/endoplasmic reticulum Ca^2+^-ATPase pumps. Also complicating the interpretation is the fact that KCNK channels are known to be targets of several second messengers. Nevertheless, our work with excised patches clearly shows that the effect of 2-APB on the members of the TREK subfamily is independent of any cytosolic factor.

It is worth noticing that 2-APB has also been used in several systems (e.g., rat dorsal root ganglia) that endogenously express KCNK10 and KCNK2 as two of their major potassium background channels (Kang and Kim, 2006). Our results should be taken into consideration when using these systems to evaluate the effect of 2-APB at concentrations higher than 300 μM. At these concentrations, 2-APB increases by nearly a factor of 2 the basal activity of hKCNK4 and hKCNK10, while also significantly enhancing the basal activity of hKCNK2 (**Figures 1G** and **3C**). Finally, elucidation of a possible common activating mechanism for members of this subfamily is deserving of further study.

## Conflict of Interest Statement

The authors declare that the research was conducted in the absence of any commercial or financial relationships that could be construed as a potential conflict of interest.
